# Lifestyle Changes Observed among Adults Participating in a Family- and Community-Based Intervention for Diabetes Prevention in Europe: The 1st Year Results of the Feel4Diabetes-Study

**DOI:** 10.3390/nu12071949

**Published:** 2020-06-30

**Authors:** Yannis Manios, Christina-Paulina Lambrinou, Christina Mavrogianni, Greet Cardon, Jaana Lindström, Violeta Iotova, Tsvetalina Tankova, Imre Rurik, Vicky Van Stappen, Jemina Kivelä, Rocío Mateo-Gallego, Luis A. Moreno, Konstantinos Makrilakis, Odysseas Androutsos

**Affiliations:** 1 Department of Nutrition and Dietetics, Harokopio University, 17671 Athens, Greece; cplambrinos@gmail.com (C.-P.L.); cmavrog@hua.gr (C.M.); 2 Department of Movement and Sports Sciences, Faculty of Medicine and Health Sciences, Ghent University, B-9000 Gent, Belgium; Greet.Cardon@UGent.be (G.C.); vivstapp.VanStappen@UGent.be (V.V.S.); 3 Department of Public Health Solutions, National Institute for Health and Welfare, 00300 Helsinki, Finland; jaana.lindstrom@thl.fi (J.L.); jemina.kivela@thl.fi (J.K.); 4 Department of Paediatrics, Medical University Varna, 9010 Varna, Bulgaria; iotova_v@yahoo.com; 5 Department of Diabetology, Clinical Center of Endocrinology, Medical University Sofia, 1431 Sofia, Bulgaria; tankova@iname.com; 6 Department of Family and Occupational Medicine, University of Debrecen, 4032 Debrecen, Hungary; rurik.imre@sph.unideb.hu; 7 Instituto Investigacion Sanitaria Aragon (IISA), CIBERCV, 50009 Zaragoza, Spain; rmateo@unizar.es; 8 Growth, Exercise, NUtrition and Development Research Group, School of Health Science (EUCS), University of Zaragoza, 50009 Zaragoza, Spain; lmoreno@unizar.es; 9 First Department of Propaedeutic Medicine, Medical School, Laiko General Hospital, National and Kapodistrian University of Athens, 11527 Athens, Greece; kmakrila@med.uoa.gr; 10 Department of Nutrition and Dietetics, School of Physical Education, Sport Science and Dietetics, University of Thessaly, 42132 Trikala, Greece; oandroutsos@uth.gr

**Keywords:** obesity, type 2 diabetes, vulnerable, families, lifestyle intervention

## Abstract

The Feel4Diabetes intervention was a school and community-based intervention aiming to promote healthy lifestyle and tackle obesity and obesity-related metabolic risk factors for the prevention of type 2 diabetes (T2D) among families at risk of developing this disease. The current study aims to present the results on lifestyle behaviors obtained from parents during the first year of the Feel4Diabetes intervention. This multicomponent intervention had a cluster randomized design and was implemented in Belgium, Bulgaria, Finland, Greece, Hungary and Spain over two years (2016–2018). Standardized protocols and procedures were used by the participating centers in all countries to collect data on parents’ lifestyle behaviors (diet, physical activity, sedentary behavior). The Feel4Diabetes intervention was registered at clinicaltrials.gov (registration number: NCT02393872). In total, 2110 high-risk parents participated in the baseline and 12-month follow-up examination measurements. Participants allocated to the intervention group reduced their daily consumption of sugary drinks (*p* = 0.037) and sweets (*p* = 0.031) and their daily screen time (*p* = 0.032), compared with the control group. In addition, participants in the intervention group in Greece and Spain increased their consumption of breakfast (*p* = 0.034) and fruits (*p* = 0.029), while in Belgium and Finland they increased their water intake (*p* = 0.024). These findings indicate that the first year of the Feel4Diabetes intervention resulted in the improvement of certain lifestyle behaviors in parents from high-risk families.

## 1. Introduction

The prevalence of type 2 diabetes (T2D) progressively increases on a global basis [1]. According to the latest report (9th edition) of the International Diabetes Federation, the number of people living with diabetes in Europe is expected to increase at least until 2045, with a large proportion of these cases remaining undiagnosed [2]. The negative impact of diabetes on public health, but also its direct and indirect financial burden on healthcare systems and society, urgently call for cost-effective interventions to halt the rise of T2D [2,3].

According to previous studies, the prevalence of T2D has been reported to be higher in Low- and Middle-Income Countries (LMICs) as well as in vulnerable population groups from High-Income Countries (HICs), including individuals with low socioeconomic status (SES) [4,5]. Therefore, public health initiatives aiming to tackle T2D should carefully consider these vulnerable population groups, starting from the early identification of people at risk using easy-to-apply, relatively low-cost and potentially scalable and sustainable screening procedures [6]. 

Previous randomized controlled trials (RCTs) have shown lifestyle interventions to be effective in improving lifestyle behaviors in individuals at high risk of developing T2D [7]. Still, given the nature of these interventions, including their intensity (frequent counseling sessions), duration (3–10 years), high demands in infrastructure and health professionals’ skills and opportunistic screening procedures (i.e., recruitment of high-risk individuals via hospitals, door-to-door procedures or by providing incentives to possible participants), they cannot be scaled up and applied to the whole population in real-life conditions. Implementing large-scale community-based interventions that target the whole population could serve this purpose, but this is not feasible due to financial and practical restrictions. Applying screening procedures to the community to target high-risk/vulnerable population groups might be more promising to tackle the increase of T2D. 

To address the aforementioned challenges, the Feel4Diabetes-study used the school setting as the entry-point to the community and the available infrastructure and personnel, when possible, to deliver a low-cost community intervention that is potentially scalable [8]. Specifically, integrating schools and community infrastructure to screen the target population, a large number of parents were screened and invited to join the study.

Although the T2D risk identification was based on parents’ FINDRISC score, the Feel4Diabetes intervention targeted the whole family. Targeting families, instead of individuals, can further support the effectiveness and cost-effectiveness of the intervention, as risk factors seem to cluster within the family, not only because the family members share a common genetic background, but also lifestyle habits, perceptions, beliefs and the social and physical environment [9,10,11].

The Feel4Diabetes intervention aimed to promote healthy eating and active lifestyle for all members of the targeted families. Face-to-face lifestyle counseling sessions were provided to the adults in the families during the first year of intervention, while during the second year the intervention was implemented via personalized SMS messages [8]. In addition, in collaboration with the local municipalities, a more supportive physical environment at schools and communities was created. 

The aim of the current work is to present the changes observed in lifestyle behaviors among the parents of high-risk families over the first year of the Feel4Diabetes intervention.

## 2. Materials and Methods

The Feel4Diabetes intervention was registered at clinicaltrials.gov (registration number: NCT02393872).

### 2.1. Ethical Approval 

The Feel4Diabetes-study adhered to the Declaration of Helsinki and the conventions of the Council of Europe on human rights and biomedicine. All participating countries obtained ethical clearance from the relevant ethical committees and local authorities. More specifically: in Belgium, the study was approved by the Medical Ethics Committee of the Ghent University Hospital (ethical approval code: B670201524437); in Bulgaria, by the Ethics Committee of the Medical University of Varna (ethical approval code: 52/10-3-2016) and the Municipalities of Sofia and Varna, as well as the Ministry of Education and Science’s local representatives; in Finland, by the hospital district of Southwest Finland’s ethical committee (ethical approval code: 174/1801/2015); in Greece, by the Bioethics Committee of Harokopio University (ethical approval code: 46/3-4-2015) and the Greek Ministry of Education; in Hungary, by the National Committee for Scientific Research in Medicine (ethical approval code: 20095/2016/EKU); and in Spain, by the Clinical Research Ethics Committee and the Department of Consumers’ Health of the Government of Aragón (ethical approval code: CP03/2016). All participants gave their written informed consent prior to their enrolment in the study.

### 2.2. Study Population

Recruitment was based on a standardized, multi-stage sampling procedure and was conducted within selected provinces in six European countries, targeting vulnerable population groups at high risk of developing T2D. In Bulgaria and Hungary (i.e., LMICs), all the municipalities within the participating regions were eligible for recruitment, while in Belgium, Finland, Greece and Spain (i.e., HICs), families within low SES municipalities were recruited. Specifically, in HICs, low SES municipalities were defined as those with the lowest educational level and/or the highest unemployment rates, as retrieved from official resources and local authorities, within each country. 

In each country, primary schools located in the selected municipalities were used as the entry-point to the community. Within the participating municipalities and schools, all families having children in the targeted classes of the first three grades of obligatory education received the “all families” component of the intervention. The families with at least one parent found to be at increased risk of T2D were invited to attend the “high-risk families” component, which was delivered out of the school setting (both components are described in the Design section below) [8]. The screening procedure followed for the identification of the high-risk adults/families has been described in detail elsewhere [6]. In brief, the identification of high-risk families was based on T2D risk estimation using the Finnish Diabetes Risk Score (FINDRISC). More specifically, “high-risk families” were considered those with at least one parent in the family fulfilling the country-specific cut-off point. The total numbers of participants in the “all families” and the “high-risk families” components are presented in Table 1. Τhe current work focuses only on the first-year results of the “high-risk families” component.

The randomization of the intervention and control group was conducted at a municipality/regional level (1:1 ratio).

### 2.3. Design

The design of the Feel4Diabetes intervention was based on the PRECEDE-PROCEED and the HAPA models, which led to the identification of the lifestyle behaviors that are associated with the risk factors to develop T2D in vulnerable population groups, as well as their determinants, which were the targets of the implemented intervention [7,8].

The duration of the intervention period was two years (2016–2018) and consisted of two components: the “all families” component delivered at schools targeting all families and the “high-risk families” component delivered out of the school curriculum and hours, in families found to be at increased risk of T2D. 

The “all families” component aimed to prevent obesity in children and promote healthy behaviors for the whole family, i.e., to: increase water consumption (instead of sugary drinks); increase consumption of fruits and vegetables; increase consumption of healthy and balanced breakfast and/or morning snack; increase physical activity; and decrease/interrupt prolonged sedentary time. The “all families” component was delivered by the teachers in the intervention schools, who were trained to deliver activities during school hours, to create a more supportive social and physical environment and promote a healthy and active lifestyle for the children. These activities were complemented with simple, easy-to-read and culturally adapted newsletters, aiming to inform and actively engage the families in the intervention. Control schools were asked to continue with the standard curriculum [8].

With regard to the “high-risk families” component, parents in the control municipalities received their medical check-up results and were offered one counseling session (or leaflet, in the case of Belgium) on lifestyle changes, which was delivered to them by trained researchers. Furthermore, they were provided with an extensive leaflet with easy to read recommendations on lifestyle changes and tips on how they could potentially introduce these changes in their own daily life as well as their children’s.

High-risk families in the intervention group, apart from the aforementioned counseling session and relevant material, received five more counseling sessions, aiming to help them to set their own specific, measurable, attainable, realistic and timely (SMART) goals, improve their self-efficacy and monitor and self-evaluate their progress over the one year period [8]. These goals were based on the general goals of the Feel4Diabetes intervention, which were identified based on a systematic literature review that was conducted in the PRECEDE-phase of the Feel4Diabetes-study and are described in Table 2.

In the school and community setting, further initiatives were taken to create a supportive physical environment for all families living in the intervention municipalities during and outside school hours, over weekdays and weekends. The main scope of these initiatives was to support and promote a more active lifestyle in the intervention municipalities.

### 2.4. Evaluation

To assess the effectiveness of the Feel4Diabetes intervention, measurements at baseline (2016) and the first follow-up (2017) were performed by well-trained researchers [12]. These measurements were conducted as close to the date of the baseline measurements as possible in both the intervention and the control group, in order to minimize the seasonality effect on participants’ behaviors. Parents’ behavioral indices were assessed by self-reported questionnaires on drinking, eating, physical activity and sedentary behaviors. The reliability of these questionnaires was evaluated prior to the study execution and was found to be acceptable [13]. 

Specifically, breakfast consumption on weekdays and on weekend days was reported in number of days, while food intake was evaluated with the use of a Food Frequency Questionnaire (FFQ), by which self-reported information on the weekly consumption of servings of different food groups was collected. For the assessment of parental sedentary behavior (i.e., on weekdays and weekend days), data were collected via the following question: “How much time do you usually spend using a computer, tablet, smartphone per day? (excluding working hours)”. In addition, the estimation of parental time spent on moderate to vigorous physical activity (MVPA) was based on relevant data collected from a short version of the International Physical Activity Questionnaire. The variables relevant to lifestyle behaviors were dichotomized based on the main Feel4Diabetes interevention’s behavioral goals.

### 2.5. Statistical analysis

Descriptive data on participants’ characteristics are presented as means or percentages for continuous or categorical variables, respectively. Comparisons of continuous variables between groups were made using Students’ T-test or the non-parametric Mann–Whitney tests, according to the normality of distribution. Pearson’s chi-square test was used in the case of categorical variables to compare percentages between groups and within the same group, with regard to changes from baseline to follow-up. The effectiveness of the intervention was further evaluated using generalized linear mixed modeling and sex as a covariate. The generalized linear mixed models provide information on the significance of the differences between the intervention and control groups in the observed mean values at baseline and follow-up (treatment effect), the within-group mean changes from baseline to follow-up (time effect) and the differences between groups with regard to these mean changes from baseline to follow-up (treatment x time interaction effect). The level of statistical significance was set as *P* ≤ 0.05, while all reported p-values were 2-tailed. Data were analyzed using the SPSS statistical analysis software, version 25.0. 

## 3. Results

The study sample comprised 2756 parents from families at high-risk of developing T2D, allocated to the intervention group (*n* = 1526) and the control group (*n* = 1230). The baseline descriptive characteristics of the total sample of study participants and by treatment arm are presented in Table 3. In brief, the study participants’ mean age was 40.9 (5.7) years, the majority were females (66.4%) and 75.5% of them had more than 12 years of education. Regarding their FINDRISC scores, 25.4% had a score that was lower than 9, 35.7% had an intermediate score from 9 to 11 and 37.1% had a score that was higher than 11. No significant differences were observed between the intervention and control groups at baseline regarding these variables.

Table 4 presents the changes from baseline to follow-up in energy-balance-related behaviors of parents by study group and by region where the study was implemented. In the total sample, a higher decrease of sugary drinks consumption was observed in the intervention compared to the control group (*p* = 0.037). Similarly, a higher decrease of sweets consumption was observed in the intervention compared to the control group (*p* = 0.031). Regarding sedentary behavior, the average time that parents reported spending on screen-related activities was found to decrease in the intervention group compared to the increase that was observed in the control group (*p* = 0.032). Furthermore, some non-significant trends in favor of the intervention group were observed, including a higher increase of breakfast (*p* = 0.287), vegetables (*p* = 0.157), fruit consumption (*p* = 0.061) and water intake (*p* = 0.069).

Additional or similar findings were observed in the statistical analyses according to the region. More specifically, in HICs under austerity measures, parents in the intervention group had a higher increase of their weekly frequency of breakfast (*p* = 0.034) and fruits (*p* = 0.029) consumption compared to the control group. Furthermore, some non-significant trends in favor of the intervention group were observed, including a higher increase of vegetables consumption (*p* = 0.061) and water intake (*p* = 0.330), as well as a higher decrease of sweets consumption (*p* = 0.150). No significant changes were observed in LMICs, but some non-significant trends in favor of the intervention group included a decrease of screen-related activities compared to an increase observed in the control group (*p* = 0.061). In HICs, an increase of water intake was observed in the intervention group compared to a decrease that was reported in the control group (*p* = 0.024). Some other, non-significant trends in favor of the intervention group included an increase of fruits consumption (*p* = 0.362), a decrease of sweets consumption (*p* = 0.229) and a decrease of screen time (*p* = 0.424).

Table 5 illustrates the proportion of study participants achieving the behavioral goals from baseline to follow-up by study group in the total sample and by region subgroups. In the total sample, a higher increase of the percentage of parents consuming ≤1 serving of sugary drinks per week was observed in the intervention group compared to the control group (*p* = 0.009). Moreover, some non-significant trends were observed in favor of the intervention group compared to the control group, such as higher increase of breakfast (*p* = 0.430) and fruit consumption (*p* = 0.525) and higher decrease of sweets consumption (*p* = 0.172) and screen time (*p* = 0.443).

In HICs under austerity measures, a significantly higher increase of the percentage of participants consuming <1 serving of sugary drinks per week was observed in the intervention group compared to the control group (*p* = 0.024). Moreover, some non-significant trends were observed in favor of the intervention group compared to the control group, such as a higher increase of breakfast (*p* = 0.225) and fruit consumption (*p* = 0.262) and a higher decrease of sweets consumption (*p* = 0.188) and screen time (*p* = 0.786). No significant changes were observed in LMICs, but some non-significant trends in favor of the intervention group included an increase of the percentage of participants consuming <1 serving of sugary drinks per week compared to a decrease that was observed in the control group (*p* = 0.064). Similarly, no significant changes were observed in HICs, but some non-significant trends were observed in favor of the intervention group compared to the control group, such as an increase of breakfast (*p* = 0.113) and fruit consumption (*p* = 0.895) and an increase of the percentage of participants consuming <1 serving of sugary drinks (*p* = 0.460) or <1 serving of sweets per week (*p* = 0.487) or devoting <2 hours per day to screen activities (*p* = 0.629) compared to decreases observed in the control group for these behaviors.

## 4. Discussion

The results of the present study showed that the Feel4Diabetes intervention improved some of the targeted dietary and sedentary behaviors of parents, especially in HICs under austerity measures (i.e., Greece and Spain). More specifically, in the total sample, the amount of energy-dense sugary drinks and sweets consumed weekly by parents was reduced, as well as the time they spent on screen-related sedentary activities (i.e., TV viewing, computer use, etc.). In HICs under austerity measures, the intervention increased the frequency of breakfast consumption and the amount of fruits and vegetables consumed by parents on a daily basis. In HICs, the intervention increased parents’ water intake. These findings are in line with previous studies that also implemented interventions targeting both children and their parents, possibly indicating that when interventions are applied to the whole family, parents are motivated to improve their dietary, sedentary and physical activity habits, in order to become role models for their children [14]. 

Moreover, previous studies focusing on the prevention of T2D via lifestyle modification in high-risk adults have showed that this approach is effective. The Finnish Diabetes Prevention Study (DPS) was an RCT promoting healthy lifestyle via dietary and physical activity counseling to middle-aged, overweight adults at high risk of developing T2D [15]. According to the findings of this study, lifestyle modification resulted in significant improvements of several determinants of T2D, such as weight loss and reduction in the prevalence of metabolic syndrome and in the prevalence of abdominal obesity, while a relative risk reduction of T2D development was achieved [15,16]. Interestingly, the study also showed that the intervention effects were sustained over a period of 13 years [17]. The Diabetes Prevention Program (DPP) was a large-scale clinical trial implemented in 27 clinical centers, involving 3000 participants in the USA and aimed at preventing T2D [18,19]. The DPP-intervention delivered an intensive lifestyle intervention via researchers who were trained on lifestyle modification, including ≥16 face-to-face, individual counseling sessions over the first 6 months of the intervention period and monthly contacts until the end of the program [18]. The results of this intervention showed favorable changes on participants’ dietary behavior and physical activity [17,20]. Similarly, the Study on Lifestyle intervention and Impaired glucose tolerance Maastricht (SLIM) delivered a 3-year lifestyle intervention in 147 adults in the Netherlands [21]. Based on the findings of this study, significant improvement of lifestyle behaviors and clinical indices (e.g., blood glucose levels, insulin resistance, free fatty acids) were observed and prevented the development of metabolic syndrome and T2D in the intervention group, with the improvements of these clinical indices being sustained after 3 years of follow-up [21,22].

Physical activity and dietary changes have been the main area of focus in T2D prevention lifestyle intervention. In the Feel4Diabetes intervention, no significant changes were observed regarding physical activity in the first year. Similar previous behavioral interventions in healthy adults did not lead to favorable changes in physical activity or were moderately effective, indicating that improving this behavior may be a complex procedure [23,24]. Individuals’ built environment may determine their physical activity levels [25]. The Feel4Diabetes-study aimed to modify high-risk families’ physical environment and provide opportunities for physical activity by co-creating activities (e.g., open school-yards in the afternoon and weekends, whole-family sport events, creating and/or informing about safe cycling routes, etc.) with local stakeholders, including mayors, municipality councils, school directors, schoolboards and parents’ associations, in the intervention municipalities. Still, it appeared that parents were not always aware of the ongoing activities and the times at which school-yards remained open for the families in the afternoon and weekends. Possibly by using new technologies and smartphone applications could instantly inform individuals of ongoing municipality activities related to physical activity, but also help them organize their own events and consequently promote changes in their physical activity behavior [26,27].

The rise of diabetes requires the design and implementation of a new generation of intervention initiatives that are easy-to-apply, scalable and sustainable, and therefore have the capacity to be transferred to the wider population. In this regard, the Feel4Diabetes-study introduced a novel approach to prevent T2D by combining school- and community-based intervention targeting both parents and children via counseling sessions and SMS-texting and by creating a more supportive environment within schools and neighborhoods. Scaling up effective interventions has been previously shown to be an effective approach to prevent T2D, especially when adaptations of the original intervention are successfully addressed in collaboration with local stakeholders to meet the local needs [28,29]. It should also be noted that a main goal during the development of the Feel4Diabetes-study was to deliver the minimum amount of counseling sessions required to effectively support individuals’ change, but also counterbalance the high costs of the initiative to achieve an optimum cost-effectiveness result. Based on the insights received from the Feel4Diabetes intervention, the participation rate in the face-to-face counseling sessions that were implemented in the first year of the intervention varied a lot and was quite low in some families (data not shown). Considering that inconvenience and lack of time constitute important barriers for attending face-to-face lifestyle interventions, for both the implementers and the users, applying electronic and mobile (e- and m-) health approaches could provide an alternative solution for delivering such interventions [30].

The findings of the present study should be interpreted in light of its strengths and weaknesses. The cluster-randomized design, the large sample size—which included participants from six European countries—and the use of standardized methods and tools for the development, implementation and assessment of the effectiveness of the Feel4Diabetes intervention are some of the strengths of the present study. On the other hand, the self-reported data collected from parents on their behaviors (i.e., dietary intake, screen time physical activity levels) are additional limitations of this study. Although the validity and reliability of the relevant questionnaires were tested before the start of the intervention, this approach is prone to recall bias and social desirability. Moreover, no data on quality of life were collected; therefore it cannot be examined if the Feel4Diabetes-study improved this parameter. Finally, the Feel4Diabetes-study did not focus on population groups following specific types of diets (e.g., vegans).

## 5. Conclusions

The current study showed that the Feel4Diabetes intervention led to some favorable changes in the dietary behavior and screen time of parents from high-risk families for T2D after the first year of the intervention. Considering that the Feel4Diabetes intervention is an easy-to-apply intervention at a relatively low cost, it could be a potentially scalable and sustainable approach to prevent T2D at the community level.

## Figures and Tables

**Table 1:** Numbers of families in the “all families” and the “high-risk families” components of the Feel4Diabetes intervention.

Country	Families Contacted	“All Families” Measured at Baseline and Follow-Up 1	“High-Risk Families” Measured at Baseline and Follow-Up 1
Belgium	5367	1502	286
Bulgaria	6541	2169	274
Finland	3247	1307	261
Greece	5195	1957	342
Hungary	2902	1684	171
Spain	4823	1448	335
Total	28075	10067	1669

**Table 2:** “Core goals” and “additional goals” of the Feel4Diabetes intervention.

CORE GOALS	ADDITIONAL GOALS
(1) Eating breakfast daily (2) ≥5 servings of vegetables every day (3) ≥3 servings of fruit or berries every day (4) <1 servings of sugary drinks per day (sodas and juices) [and drink water/coffee instead] (5) ≥150 min of moderate to vigorous physical activity per week (6) <120 min of screen time (excluding work and school) per day	(7) ≥4 servings of whole-grain foods per day (8) ≤1 servings of sweets, biscuits, ice cream, cakes, pastries per week (9) ≤1 servings of salty snacks/fast food per week (hamburgers, chips, pizza, savory pastries, etc.) (10) ≥3 servings (3 × 30 g) of nuts per week (11) ≤2 servings of red and/or processed meat per week (12) ≥1 serving of low-fat dairy products per day (13) Use of olive or rapeseed oil or soft margarines (14) Weight loss of >5% of the baseline body weight, for those who are overweight/obese (15) Family meal at table once a day

**Table 3:** Descriptive characteristics of study participants in the total sample and by study group.

	Total Sample	Control Group	Intervention Group	*p-*Value *
	(*n* = 2756)	(*n* = 1230)	(*n* = 1526)	
Age	40.9 (5.66)	40.9 (5.81)	41.0 (5.55)	0.221 §
Females, % (*n*)	66.4 (1830)	66.6 (819)	66.3 (1011)	0.854 ‡
Educational level, % (*n*)				
≤12 years	24.5 (676)	26.0 (320)	23.3 (356)	0.103 ‡
>12 years	75.5 (2080)	74.0 (910)	76.7 (1170)	
FINDRISC categories, % (*n*)				
<9	25.4 (700)	25.0 (307)	25.8 (393)	0.424 ‡
9–11	37.5 (1033)	36.6 (450)	38.2 (583)	
>11	37.1 (1023)	38.5 (473)	36.0 (550)	
FINDRISC value	10.3 (4.20)	10.4 (4.24)	10.3 (4.17)	0.398 §

Data are presented as means (SD), percentages (%) and frequencies (n). Age is presented in years. FINDRISC: Finnish Diabetes Risk Score. * *p*-values indicate the significance of the differences between study groups. ‡ *p* values were derived from the chi-square test, § *p* values were derived from the nonparametric Mann–Whitney test.

**Table 4:** Changes from baseline to follow-up in energy-balance-related behaviors of parents by study group, in the total sample and in each region/country.

	Total Sample					HICs under Austerity Measures					LMICs					HICs				
	Baseline		Follow-Up		*p-*Value *	Baseline		Follow-Up		*p-*Value *	Baseline		Follow-Up		*p-*Value *	Baseline		Follow-Up		*p-*Value *
**Breakfast (days per week)**	**mean**	**SE**	**mean**	**SE**		**mean**	**SE**	**mean**	**SE**		**mean**	**SE**	**mean**	**SE**		**mean**	**SE**	**mean**	**SE**	
Intervention	5.34	1.62	5.62	1.62	**0.003**	5.56	1.55	6.06	1.56	**<0.001**	4.54	1.74	4.50	1.74	0.809	5.94	1.23	6.16	1.23	0.134
Control	5.36	1.62	5.49	1.62	0.180	5.33	1.56	5.42	1.56	0.549	4.47	1.74	4.65	1.74	0.409	6.22	1.23	6.20	1.23	0.919
*p-value* §	0.790		0.249		0.287 ‡	0.079		**<0.001**		**0.034** ‡	0.671		0.507		0.433 ‡	**0.025**		0.752		0.241 ‡
**Water (ml per day)**																				
Intervention	1101.5	514.2	1182.6	514.4	**0.007**	1167.5	528.5	1266.6	528.9	**0.025**	1137.9	493.2	1125.0	494.0	0.812	920.8	498.3	1062.8	499.3	**0.017**
Control	1076.1	514.3	1077.4	514.4	0.966	1157.7	528.7	1192.2	529.1	0.485	1068.9	493.6	1085.0	494.7	0.798	937.8	498.4	896.4	498.8	0.454
*p-value* §	0.360		**0.002**		0.069 ‡	0.818		0.144		0.330 ‡	0.173		0.542		0.726 ‡	0.741		**0.008**		**0.024** ‡
**Sugary drinks (ml per day)**																				
Intervention	78.8	106.6	53.7	106.6	**<0.001**	51.9	80.3	24.4	80.4	**<0.001**	119.7	138.8	98.1	139.2	0.197	87.6	105.1	75.7	105.3	0.353
Control	83.9	106.6	78.7	106.6	0.460	65.0	80.4	46.2	80.4	**0.017**	112.4	139.0	134.0	139.3	0.247	93.1	105.2	92.4	105.3	0.954
*p-value* §	0.400		**0.001**		**0.037** ‡	0.055		**0.007**		0.412 ‡	0.632		0.071		0.084 ‡	0.620		0.223		0.527 ‡
**Vegetables (servings/day)**																				
Intervention	1.13	0.78	1.24	0.79	**0.011**	1.09	0.82	1.33	0.82	**<0.001**	1.02	0.76	1.05	0.76	0.761	1.35	0.73	1.33	0.73	0.752
Control	1.06	0.78	1.08	0.79	0.657	0.99	0.82	1.05	0.82	0.479	1.01	0.76	0.98	0.96	0.771	1.20	0.73	1.20	0.73	0.946
*p-value* §	0.080		**0.001**		0.157 ‡	0.145		**<0.001**		0.061 ‡	0.813		0.474		0.674 ‡	**0.043**		0.158		0.852 ‡
**Fruits (servings/day)**																				
Intervention	1.05	0.73	1.17	0.73	**0.004**	1.07	0.78	1.37	0.78	**<0.001**	1.02	0.66	0.88	0.66	**0.042**	0.99	0.70	1.05	0.70	0.417
Control	0.93	0.73	0.94	0.73	0.894	0.93	0.78	1.01	0.78	0.221	0.81	0.66	0.69	0.66	0.139	1.00	0.70	0.96	0.70	0.643
*p-value* §	**0.003**		<0.001		0.061 ‡	**0.022**		**<0.001**		**0.029** ‡	**0.002**		**0.032**		0.824 ‡	0.879		0.293		0.362 ‡
**Sweets (servings/day)**																				
Intervention	0.55	0.47	0.43	0.47	**<0.001**	0.59	0.49	0.42	0.49	**<0.001**	0.49	0.46	0.44	0.46	0.348	0.57	0.45	0.44	0.45	**0.014**
Control	0.56	0.47	0.52	0.47	0.180	0.54	0.49	0.46	0.49	0.080	0.53	0.46	0.56	0.46	0.596	0.63	0.45	0.58	0.45	0.375
*p-value* §	0.710		**0.002**		**0.031** ‡	0.256		0.353		0.150 ‡	0.447		0.060		0.308 ‡	0.199		**0.009**		0.229 ‡
**Screen time (hours per day)**																				
Intervention	3.64	1.22	3.48	1.22	**0.034**	3.59	1.21	3.39	1.22	**0.045**	3.70	1.30	3.62	1.30	0.560	3.64	1.15	3.55	1.15	0.506
Control	3.67	1.22	3.74	1.22	0.346	3.61	1.22	3.55	1.22	0.630	3.66	1.30	3.98	1.31	**0.049**	3.76	1.15	3.82	1.15	0.646
*p-value* §	0.633		**0.002**		**0.032** ‡	0.900		0.165		0.322 ‡	0.754		**0.033**		0.061 ‡	0.307		0.063		0.424 ‡
**MVPA (minutes per week)**																				
Intervention	325.6	315.4	311.4	315.5	0.474	307.6	322.3	277.4	322.6	0.291	377.2	339.7	396.7	340.6	0.638	299.3	277.3	290.8	277.9	0.815
Control	328.3	315.6	331.5	315.6	0.878	354.8	322.4	322.9	322.8	0.320	325.4	340.1	362.6	341.1	0.437	287.7	277.3	323.2	277.5	0.282
*p-value* §	0.884		0.374		0.547 ‡	0.082		0.172		0.969 ‡	0.171		0.501		0.779 ‡	0.709		0.395		0.371 ‡

MVPA: Moderate to vigorous physical activity; HICs: High-income countries; LMICs: Low- to middle-income countries. Serving size: for fruits and vegetables: 1/2 cup, for sweets: one small chocolate bar (40 g) or half a cup of sweets, cookies or one scoop of ice cream. * *p*-values indicate the time effect and were derived from generalized linear mixed modeling with sex as a covariate. § *p*-values indicate the treatment effect and were derived from generalized linear mixed modeling with sex as a covariate. ‡ *p*-values indicate the treatment × time interaction effect and were derived from generalized linear mixed modeling with sex as a covariate. Significant *p*-values are highlighted in bold.

**Table 5:** Changes from baseline to follow-up in the percentage of parents achieving the behavioral goals by study group in the total sample and in each region/country.

		Total Sample			HICs under Austerity Measures			LMICs			HICs		
		Baseline	Follow-Up	*p*-Value *	Baseline	Follow-Up	*p*-Value *	Baseline	Follow-up	*p*-Value *	Baseline	Follow-Up	*p*-Value *
Breakfast		%	%		%	%		%	%		%	%	
Intervention	daily	57.8	61.6	0.062	65.2	71.7	**0.019**	35.2	31.0	0.242	71.0	77.7	0.071
Control	daily	60.0	61.5	0.478	62.8	64.3	0.651	33.9	33.2	0.855	79.1	77.9	0.708
	*p-*value §	0.245	0.971	0.430 ‡	0.381	**0.019**	0.225 ‡	0.703	0.613	0.533 ‡	**0.010**	0.965	0.113 ‡
**Sugary drinks**		%	%		%	%		%	%		%	%	
Intervention	<1 serving per week	54.6	66.4	**<0.001**	59.5	77.0	**<0.001**	46.4	53.1	0.117	86.9	90.5	0.196
Control	<1 serving per week	52.3	56.3	0.087	54.6	63.8	**0.007**	50.3	45.8	0.336	88.2	87.7	0.850
	*p-*value §	0.254	**<0.001**	**0.009** ‡	0.104	**<0.001**	**0.024** ‡	0.305	0.152	0.064 ‡	0.607	0.330	0.460 ‡
**Vegetables**		%	%		%	%		%	%		%	%	
Intervention	>=5 servings per day	3.4	2.8	0.387	3.6	3.2	0.701	3.8	2.9	0.537	2.6	1.8	0.526
Control	>=5 servings per day	2.7	3.4	0.298	3.2	4.3	0.375	4.1	4.2	0.937	0.5	1.7	0.144
	*p-*value §	0.245	0.430	0.623 ‡	0.701	0.387	0.595 ‡	0.838	0.447	0.856 ‡	**0.023**	0.927	0.719 ‡
**Fruits**		%	%	%	%	%		%	%	%	%	%	
Intervention	>=3 servings per day	9.4	11.7	0.063	10.4	16.7	**0.001**	8.5	4.0	**0.021**	8.6	10.5	0.448
Control	>=3 servings per day	7.0	7.5	0.648	7.4	9.0	0.358	5.2	2.3	0.095	8.0	9.2	0.581
	*p-*value §	**0.020**	**0.002**	0.525 ‡	0.060	**0.001**	0.262 ‡	0.078	0.303	0.780 ‡	0.750	0.622	0.895 ‡
**Sweets**		%	%	%	%	%		%	%	%	%	%	
Intervention	<= 1 serving per week	24.1	28.0	**0.026**	24.5	31.3	**0.009**	25.7	25.8	0.980	21.2	23.6	0.485
Control	<= 1 serving per week	23.5	23.6	0.954	28.5	29.8	0.662	19.9	20.1	0.952	19.1	17.7	0.644
	*p-*value §	0.708	0.028	0.172 ‡	0.104	0.631	0.188 ‡	0.051	0.138	0.973 ‡	0.470	0.097	0.487 ‡
**Screen time**		%	%	%	%	%		%	%	%	%	%	
Intervention	<2 h per day	16.6	18.5	0.219	17.9	21.0	0.193	19.1	19.4	0.928	11.0	12.0	0.700
Control	<2 h per day	14.9	14.6	0.842	16.2	18.1	0.430	19.4	16.9	0.470	8.8	8.0	0.715
	*p-*value §	0.222	**0.024**	0.443 ‡	0.410	0.294	0.786 ‡	0.926	0.486	0.630 ‡	0.320	0.132	0.629 ‡
**MVPA**		%	%	%	%	%		%	%	%	%	%	
Intervention	>=150 min per week	46.9	47.6	0.756	42.3	43.6	0.692	51.5	53.4	0.653	50.6	50.0	0.892
Control	>=150 min per week	46.5	47.5	0.666	43.0	44.7	0.638	49.3	52.2	0.555	49.5	48.4	0.793
	*p-*value §	0.858	0.989	0.909 ‡	0.818	0.764	0.876 ‡	0.582	0.825	0.863 ‡	0.780	0.745	0.914 ‡

MVPA: Moderate to vigorous physical activity; HICs: High-income countries; LMICs: Low- to middle-income countries. Serving size: for sugary drinks: 250 mL, for fruits and vegetables: 1/2 cup, for sweets: one small chocolate bar (40 g) or half a cup of sweets, cookies or one scoop of ice cream. * *p*-values indicate the time effect and were derived from generalized linear mixed modeling with sex as a covariate. § *p*-values indicate the treatment effect and were derived from generalized linear mixed modeling with sex as a covariate. ‡ *p*-values indicate the treatment × time interaction effect and were derived from generalized linear mixed modeling with sex as a covariate. Significant *p*-values are highlighted in bold.
